# Decoding the functional diversity of plant growth-promoting bacterial communities in the soils of Western Ghats, Tamil Nadu, India

**DOI:** 10.3389/fmicb.2026.1865342

**Published:** 2026-08-21

**Authors:** Manikandan Murugesan, Sugitha Thankappan, Vellaichamy Mageshwaran, Rajendran Ramasamy, Arvinth Singaram

**Affiliations:** 1 Department of Botany, PSG College of Arts & Science, Coimbatore, Tamil Nadu, India; 2 Department of Agricultural Engineering, SNS College of Technology, Coimbatore, Tamil Nadu, India; 3 Microbial Technology Laboratory, ICAR-National Bureau of Agriculturally Important Microorganisms, Mau, Uttar Pradesh, India; 4 Department of Microbiology, PSG College of Arts & Science, Coimbatore, Tamil Nadu, India

**Keywords:** antifungal activity, bacterial diversity, chickpea, metagenomics, plant growth promotion, Western Ghats

## Abstract

Western ghats in India, one of the world’s biodiversity hot spots is the reservoirs of microbial resources having agricultural and industrial significance. However, the diversity of plant growth-promoting microbial communities associated with the plants and soil in the Western Ghats is untapped vault. The current emphasis on natural farming is more depending on the indigenous microbial communities and their metabolic functions towards sustainable one -health. With this background, the present study examines the bacterial diversity of soils from the Western Ghats of Nilgiris, Coimbatore and Dindigul regions. Among the 10 soil samples collected (S1 to S10), three soil samples (S2, S4 and S6) representing respective three regions were subjected for metagenomic studies based on their distinct soil chemical and biological properties. The computational analysis of the metagenome revealed the core genus *Bradhyrhizobium* in all soil samples, while *Trebonia*, *Arthrobacter, Streptomyces,* and *Pseudomonas* are the next most abundant genera, which varied substantially. The results collectively demonstrate that soil sample from Dindigul harbours the richest and most diverse microbial community among the three regions. In culturable studies, a total of 101 bacterial isolates were obtained from 10 soil samples (S1 to S10). Among them four Gram-negative bacterial isolates showed potential plant growth-promoting attributes, such as Ammonia, Indole Acetic Acid, Hydrogen cyanide and siderophore production, phosphorus, potassium, and zinc solubilization. The 16S rDNA analysis revealed that the bacterial isolates were *Pseudomonas glycinae* S6B1, *Pseudomonas tolaasii* S2B3, *Pseudomonas azotoformans* S9H10, and *Pseudomonas poae* S10B2. The isolate, S10B2, exhibited the maximum inhibition, with 81.25%, 70.1%, and 35% against plant pathogenic fungi, *Rhizoctonia solani*, *Sclerotium rolfsii*, and *Fusarium oxysporum*, respectively, indicating strong biocontrol potential. The effect of bacterial inoculants on chick pea (*Cicer arietinum* var. JG 62), showed that *P. glycinae* S6B1 significantly promoted plant growth such as root length, shoot length, and fresh/dry biomass. These findings unlock the core microbiome of soils of Western Ghats, which can be utilized to develop a synthetic microbial consortium to boost agricultural productivity.

## 1 Introduction

Soil is a living ecosystem, and its productivity depends on the presence of its microbial inhabitants, biological activity, and diversity. Soil microorganisms, including bacteria, fungi, actinomycetes, protozoa, archaea, and viruses, play crucial roles in sustaining ecological functions such as organic matter turnover, nutrient cycling, pollutant detoxification, and maintaining soil structure (Singh et al., 2011). Among these, bacteria and fungi are dominant in number and function, acting as crucial mediators in carbon, nitrogen, phosphorus, and sulphur cycling. As global agriculture intensifies to meet the growing demands of the population, preserving and utilizing this microbial wealth has become paramount to sustainable soil management and crop productivity (Kumar, 2024).

Microbial diversity is frequently regarded as a cornerstone of soil health, influencing the system’s ability to withstand and recover from natural or anthropogenic disturbances (Ogwu et al., 2024). Diverse microbial communities provide functional redundancy, enabling critical soil processes to persist even when individual taxa are absent. These microbes secrete various extracellular enzymes that degrade intricate organic materials into simpler compounds, which plants subsequently mineralize and assimilate. Furthermore, microbial residues contribute to the formation of humus and stable soil aggregates, enhancing soil aeration, moisture retention, and erosion resistance (Abdelhak, 2022).

The increasing reliance on synthetic agrochemicals in conventional agriculture has led to a significant rise in environmental pollution. Several unintended consequences, including the depletion of beneficial microbial populations, nutrient leaching, soil compaction, and contamination of water bodies, have resulted. The continuous application of chemical fertilizers leads to microbial dysbiosis, resulting in the soil microbiome shifting toward less resilient or less functional communities (Verma et al., 2017). The Western Ghats in India is one of the world’s biodiversity hotspots. Indigenous microbial communities have adapted to local soil and climatic conditions (Ghare et al., 2023). They have developed mechanisms to endure salinity, acidity, nutrient scarcity, and heavy metal stress. The Western Ghats is relatively undisturbed due to less anthropogenic disturbance and harbors a pool of rhizospheric microorganisms that can be leveraged for ecologically responsible farming practices (Shabeer Ali et al., 2023). However, these unique microbial ecosystems remain underexplored and underutilized in mainstream agriculture.

Plant Growth-Promoting Rhizobacteria (PGPR) can enhance the plant growth through direct and indirect mechanisms (Kapoor et al., 2024). Direct mechanisms include biological nitrogen fixation, phosphate solubilization, potassium mobilization, and the biosynthesis of phytohormones such as indole-3-acetic acid (IAA), cytokinins, and gibberellins. Indirect mechanisms involve the suppression of soil-borne pathogens through the production of siderophores, antibiotics, hydrogen cyanide (HCN), and induced systemic resistance (ISR) in plants (Olanrewaju et al., 2017). The application of PGPR has been demonstrated to significantly enhance seed germination, root elongation, chlorophyll content, and crop yield across diverse agricultural systems. However, the culturable bacterial diversity is limited owing to symbiotic partners, crucial nutrient requirements in lab protocols and complex environmental factors (Lewis et al., 2021). The unculturable diversity studied through next-generation sequencing provides insights on the untapped microbiome, novel genes and metabolic pathways.

In agricultural perspective, to combat climate resilience, NGS especially Metagenomic approach explores unknown plant growth-promoting microbes, stress-mitigating gene clusters, and biodegradation pathways, offering environmentally compatible alternatives to chemical pesticides and fertilizers. Incorporating metagenomic data into predictive models facilitates the development of climate-resilient cropping systems, bioremediation strategies, and conservation approaches for sustainable farming (Roohi et al., 2025).

Chickpea (*Cicer arietinum* L.) is a food legume crop of universal importance as a protein source to millions of populations. It improves the soil nutritional status and health by symbiotic nitrogen fixation (Jendoubi et al., 2017). It plays a crucial role in ensuring food security, particularly in rainfed and semi-arid environments. However, the crop is more prone to several biotic and abiotic stresses (Parmar and Gohel, 2021). Studies showed that inoculation of *Mesorhiobium* strains associated with PGPR can significantly enhance chickpea productivity and improved the root nodulation, biomass accumulation and nitrogen fixation (Kumar et al., 2025; Ryan et al., 2008: Senthilkumar et al., 2009). The seed biopriming with plant growth promoting bacteria has induced the plant growth and systemic resistance and mitigates the biotic stress in chickpea plants (Mageshwaran et al., 2022, 2026; Elbouazaoui et al., 2022). The present study aims to understand the functional diversity of plant growth promoting bacterial communities in soils of Western Ghats of Tamil Nadu and its effect on chickpea growth.

## 2 Materials and methods

### 2.1 Collection of soil samples

**Figure 1:**
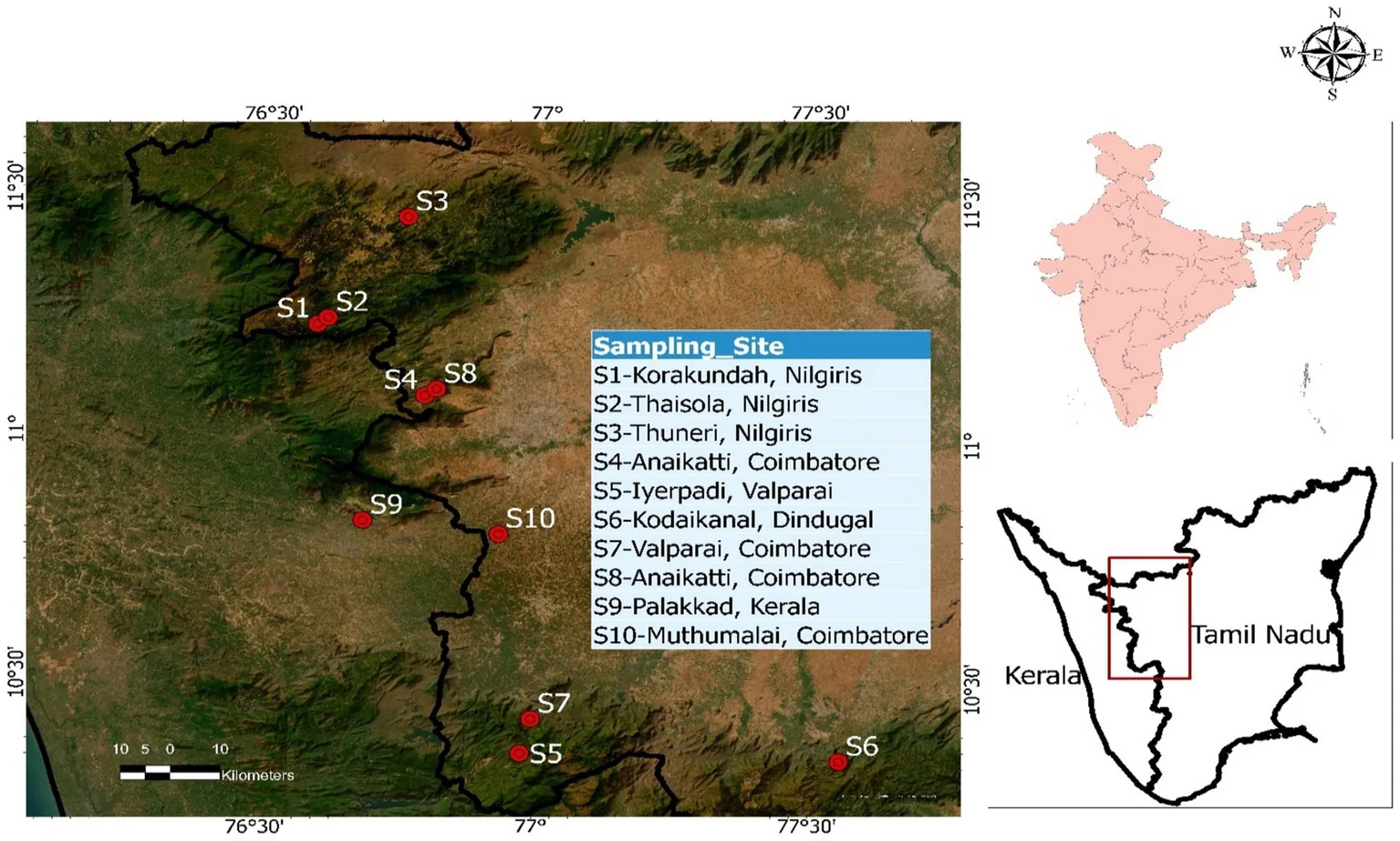
Sampling sites of different regions of the Western Ghats, India. Reproduced from Map data ©2026 Google.

A total of 10 soil samples were collected aseptically from different sites (S1 to S10) of Western Ghats of Tamil Nadu, India (Figure 1). The sites represented the semi-forested soils with low visible anthropogenic disturbance of three regions viz., Nilgiris, Coimbatore and Dindugul. Soil samples were obtained from the rhizospheric layer using sterile spatula, placed in sterile containers, transported to the laboratory under cooled conditions and processed immediately. At the time of sampling, longitude and latitude, soil pH and type of vegetation were recorded (Table 1).

**Table 1:** Soil sampling details.

Soil sampling site	District	Soil pH	Latitude	Longitude	Vegetation
Site 1- Korakundah (S1)	Nilgiris	4.58	11°13′43”	76°34′84”	*Camellia sinensis*
Site 2-Thaisola (S2)	Nilgiris	5.05	11°13′92”	76°36′32”	*Camellia sinensis*
Site 3-Thuneri (S3)	Nilgiris	5.82	11°27′10”	76°44′62”	*Ageratina adenophora*, *Eucalyptus globulus*, *Maesa indica*,
Site 4-Anaikatti (S4)	Coimbatore	7.20	11°05′08”	76°46′80”	*Ageratum conyzoides, Ziziphus xylopyrus, Psydrax umbellata,*
Site 5- Valparai (S5)	Coimbatore	4.72	10°21′14”	76°58′35”	*Camellia sinensis*
Site 6-Kodaikanal (S6)	Dindugul	5.95	10°20′49”	77°33′19”	*Cassia occidentalis, Turpinia nepalense, Argyreia cuneta*
Site 7- Valparai (S7)	Coimbatore	4.98	10°25′29”	76°59′41”	*Camellia sinensis*
Site 8- Anaikatti (S8)	Coimbatore	6.40	11°05′57”	76°47′95”	*Lantana camera*
Site 9-Palakkad (S9)	Kerala	6.20	10°49′36”	76°40′52”	*Pongamia pinnata, Hugonia serrata, Ehretia laevis*
Site 10- Muthumalai (S10)	Coimbatore	7.10	10°47′72”	76°55′45”	*Sida acuta, Alangium salvifolium, Gymnema sylvestre.*

### 2.2 Microorganisms and culture conditions

The soil-borne fungal pathogens, viz., *Rhizoctonia solani, Sclerotium rolfsii*, and *Fusarium oxysporum*, were obtained from Microbial Technology Laboratory, ICAR-NBAIM, Mau. The fungal cultures were maintained in potato dextrose agar (PDA) (Himedia, India) at 30 °C ± 2 °C for 96 h. The bacterial cultures obtained in the study were grown in nutrient agar (NA) (Himedia, India) at 30 °C ± 2 °C. The fungal and bacterial cultures were maintained at 4 °C till further use.

### 2.3 Analysis of soil chemical and biological properties

The soil chemical properties such as Organic C, Total N, Available P and K and the soil biological properties such as dehydrogenase, cellulase and phosphatase activities were determined according to Genova et al. (2024).

### 2.4 DNA isolation, library preparation, and metagenomic sequencing

The soil samples having distinct chemical and biological properties viz., S2, S4 and S6 representing the regions of Nilgiris, Coimbatore, and Dindugul, was named as sample 1, 2 and 3, respectively for the metagenomic study. Genomic DNA was extracted from three soil samples using the DNeasy PowerSoil Pro Kit (Cat. No. 47014), and the DNA concentration was estimated using a Qubit 4.0 fluorometer. The extracted genomic DNA was then amplified by universal 16S rRNA primers (8F and 1492R) and analyzed on a 1.8% agarose gel. A total of 25 ng DNA was used to generate a paired-end library preparation using the Twist NGS Library Preparation Kit for Illumina according to manufacturer protocol. The amplified libraries were subsequently analysed on an Agilent Tape Station 4150 equipped with High Sensitivity D1000 ScreenTape and sequenced on Illumina NovaSeq X Plus platform.

Metagenomic raw reads were obtained from the Illumina Novaseq platform were quality filtered using Fastp v0.6.4_Dev (Chen et al., 2018) to remove the adaptor sequences and low-quality reads, the filtered reads were assembled with denovo using MEGAHIT V1.2.9 (Li et al., 2015). The predicted protein coding genes were assembled from the contigs using Prodigal V2.6.3 (Hyatt et al., 2010). Taxonomic classification reads were performed using Kaiju databases 1.9.0 (Menzel et al., 2016). Functional annotation of the predicted genes was carried out using KEGG, COG and eggnog database through eggnog-mapper v2.1.12 pipeline (Cantalapiedra et al., 2021). Alpha and Beta diversity was performed using QIIME 2 software (Bolyen et al., 2019). Antimicrobial resistance was identified using the predicted protein sequences against the CARD database using v2.13.0+ (Alcock et al., 2023).

### 2.5 Isolation and enumeration of culturable bacteria

The stock of the soil samples was prepared by adding 1 g of the soil samples to 10 mL of sterile distilled water in a conical flask. The samples were serially diluted up to 10^−8^. One mL of the enumerated soil samples was plated on nutrient agar, Luria-Bertani agar, and R2-A agar media (Himedia, India) and incubated for 24 to 48 h at 30 °C ± 2 °C. Single colonies having distinct morphotypes were picked and streaked on sterile nutrient agar plates. A total of 101 bacterial isolates were obtained and used for further screening. The bacterial population was enumerated and expressed as colony-forming units (CFU) per g of soil as follows.


$$\mathrm{CFU}/g\;\text{of soil}=\frac{\text{Number of colonies}\times \text{Dilution factor}}{\text{Weight of soil sample}\;(g)}$$

### 2.6 Characterization of plant growth-promoting (PGP) traits

The PGPR qualitative traits of bacterial isolates grown in nutrient broth for 24 h were characterized using methods adapted from Sharma et al. (2017) with slight modifications (Singh et al., 2017). The plant growth-promoting traits of bacterial isolates were evaluated using standard assays.

The phosphate-solubilizing ability of bacterial isolates was evaluated on Pikovskaya agar medium (Devi et al., 2023). Potassium solubilization was assessed on Aleksandrow agar medium containing 0.2% potassium alumino-silicate, as described by Joe et al. (2018). The zinc solubilization ability of bacterial isolates was evaluated on zinc solubilization agar medium prepared with ZnO (1 g/L), following the method of Saravanan et al. (2004). Bacterial isolates (10 μL) were spot-inoculated and incubated at 30 °C ± 2 °C for 72 h, where halo zone formation confirmed positive activities for P, K and Zn. Ammonia production was confirmed in peptone broth cultures treated with Nessler’s reagent, which produced a pale yellow to dark brown coloration (Cappuccino and Sherman, 2002).

Siderophore production was determined by the (CAS) agar assay of Schwyn and Neilands (1987). Hydrogen cyanide production was assessed on nutrient agar supplemented with 0.44% glycine, as described by Marag and Suman (2018). Bacterial cultures were streaked, and picric acid–impregnated filter paper was placed in tubes and incubated at 30 °C ± 2 °C for 48 h. A yellowish to reddish-brown colour indicated positive HCN production. Indole-3-acetic acid (IAA) production was quantified using the colorimetric method described by Mohite (2013). Cultures grown in nutrient broth with 0.1% L-tryptophan were processed with Salkowski’s reagent.

One hundred and one culturable bacterial isolates were assessed based on the qualitative plant growth-promoting (PGP) traits using scoring system (Mageshwaran et al., 2022). The scoring was based on the following four categories: absent, low, medium, and high with the value of 0, 1, 2 and 3, respectively. In potassium, phosphorus, zinc and siderophore assays, the scoring system was done based on diameter (mm) of halo zone formation with 5–8, 9 to 12, and >12 corresponds to low, medium, and high, respectively. The ammonia and Hydrogen cyanide (HCN) production was evaluated according to the change in colour of the indicator, where pale yellow, yellow and yellowish-brown and corresponds to low, medium, and high, respectively. The IAA production was assessed based on colour intensity, where pale pink, pink, and pinkish-red indicated low, medium, and high, respectively. Based on the overall scores obtained for all PGP traits, the best-performing isolates were selected for further study.

### 2.7 Biochemical characterization of selected plant growth-promoting bacterial isolates

The selected bacterial isolates (S2B3, S10B2, S6B1, S9H10) were characterized using standard microbiological and biochemical tests according to Dubey (2008).

### 2.8 Quantitative determination of antifungal property of selected plant growth-promoting bacterial isolates

The bacterial isolates (S2B3, S10B2, S6B1, S9H10) were estimated quantitatively for antifungal property by dual culture assay. The fungal disc of 5 mm was kept at the centre of a fresh PDA plate, and the selected bacterial isolates were streaked (co-inoculated) on both sides of the Petri plates equidistant from the disc and incubated at 30 °C ± 2 °C for 48 h. A control was maintained in which the bacteria were not inoculated. The inhibition percentage was calculated using the formula:


$$\begin{array}{l} \text{Percent inhibition using the Dual culture assay}=\\ \frac{\text{Mycelial growth of fungal pathogen in control}-\text{Mycelial growth in treated}}{\text{Mycelial growth in control}\;}\times 100 \end{array}$$

### 2.9 Quantitative determination of potassium solubilization of selected plant growth-promoting bacterial isolates

Bacterial culture (0.5 mL) was inoculated with 50 mL of Aleksandrow broth containing 0.2% potassium aluminum-silicate. The cultures were incubated for 5 days at 30 °C ± 2 °C in a rotary shaker to assess bacterial growth. The obtained filtrate was centrifuged at 3500 rpm for 5 min; then the pellet was discarded. Potassium solubilization in the supernatant was quantified using a Flame Photometer. The uninoculated broth was used as the control. The solubilization rate (μg/ml) was determined by taking the difference between the values of the treated and the control.

### 2.10 Molecular identification of selected plant growth-promoting bacterial isolates

The selected Bacterial isolates were grown in Nutrient Broth at 30 °C ± 2 °C for 24 h, centrifuged, and the pellets were separated. The pellets were used for DNA extraction. Genomic DNA from selected bacterial strains was obtained by using a DNA isolation kit (Nucleo-pore, Genetic Biotech Asia Pvt. Ltd.). The amplification of the 16S rRNA gene was carried out with universal primers 27F (AGA GTT TGA TCCTGGCTCAG) and reverse primer 1,492 R (TAC GGT TAC CTT GTT ACGACT), GeNei, India (Weisburg et al., 1991). The PCR amplification for 16S rRNA (35 cycles) was performed using a thermocycler (Thermo Fisher Scientific, United States). The amplicon (~1,500 bp) was purified with Sure Extract PCR clean-up kit (Nucleo-pore) and sequenced (ABI 3130XL, Applied Biosystems), and the sequence quality was checked using Finch TV software. The partial 16S rRNA gene sequences were used to perform BLAST against available sequences in NCBI to identify their closest neighbors. ClustalW and the neighbor-joining method (Thompson et al., 1994) were employed for multiple sequence alignment and phylogenetic tree construction, respectively, using MEGA 11 (Tamura et al., 2021).

### 2.11 Evaluation of selected plant growth-promoting bacterial isolates on the growth of chick pea

The effect of selected bacteria (S2B3, S10B2, S6B1, and S9H10) on the growth promotion of chickpea was evaluated by soft agar plate technique, paper towel method, and pot culture trials. A sterile 90 mm Petri plate was filled with 20 mL of 1% agar solution. Healthy chickpea seeds (*Cicer arietinum L*.) were collected, surface sterilized and washed thoroughly 4–5 times with sterile-distilled water, and air-dried aseptically with germination paper (Mageshwaran et al., 2022). The seed bacterization was done in which surface sterilized seeds were immersed in 24 h old culture of bacterial isolate grown in nutrient broth with the population of 1.25 × 10^5^ CFU ml^−1^ and kept for 30 min incubated under laboratory conditions. A control was maintained in which no bacteria was treated to the chick pea seeds. The seeds were soaked in the bacterial suspension for 30 min prior to sowing. In the agar plate technique, the treated seeds (5 numbers) were placed in Petri plates and incubated in a BOD incubator at 30 °C ± 2 °C for 14 days. For the paper towel method, the treated seeds were kept in a paper towel and incubated for 14 days. For the pot culture study, the experimental soil was collected from the agricultural farm, ICAR-NBAIM, Mau. Clean plastic pots (9 × 15 cm) were three-quarters filled with sterile soil. Each pot contained approximately 2.5 to 3 kg of sterile soil. Five treated seeds were sown at a depth of 4 to 5 cm in the soil of each pot. In all experiments, a control was maintained in which the seeds were immersed in plain nutrient broth for 30 min, and observations of the pot culture experiment were taken after 28 days. After 28 days, chickpea plants were uprooted, and soil particles stuck on the roots were washed with a glass tray filled with water. The plant growth parameters, including root and shoot lengths (cm) and fresh and dry biomass (g), were measured. Root and shoot dry weights were measured following 24 h of drying at 70 °C in a hot air oven. In all the experiments, triplicate samples were kept under each treatment.

### 2.12 Statistical analysis

The experiments were laid out with three and five replications, data analysis was recorded using Microsoft Excel, subjected to variance analysis (ANOVA), followed by Duncan’s multiple range test and Tukey’s HSD *post hoc* test using SPSS software. Results were graphically represented and tabulated as mean ± standard deviation (SD).

## 3 Results

### 3.1 Chemical and biological properties of soil

**Figure 2:**
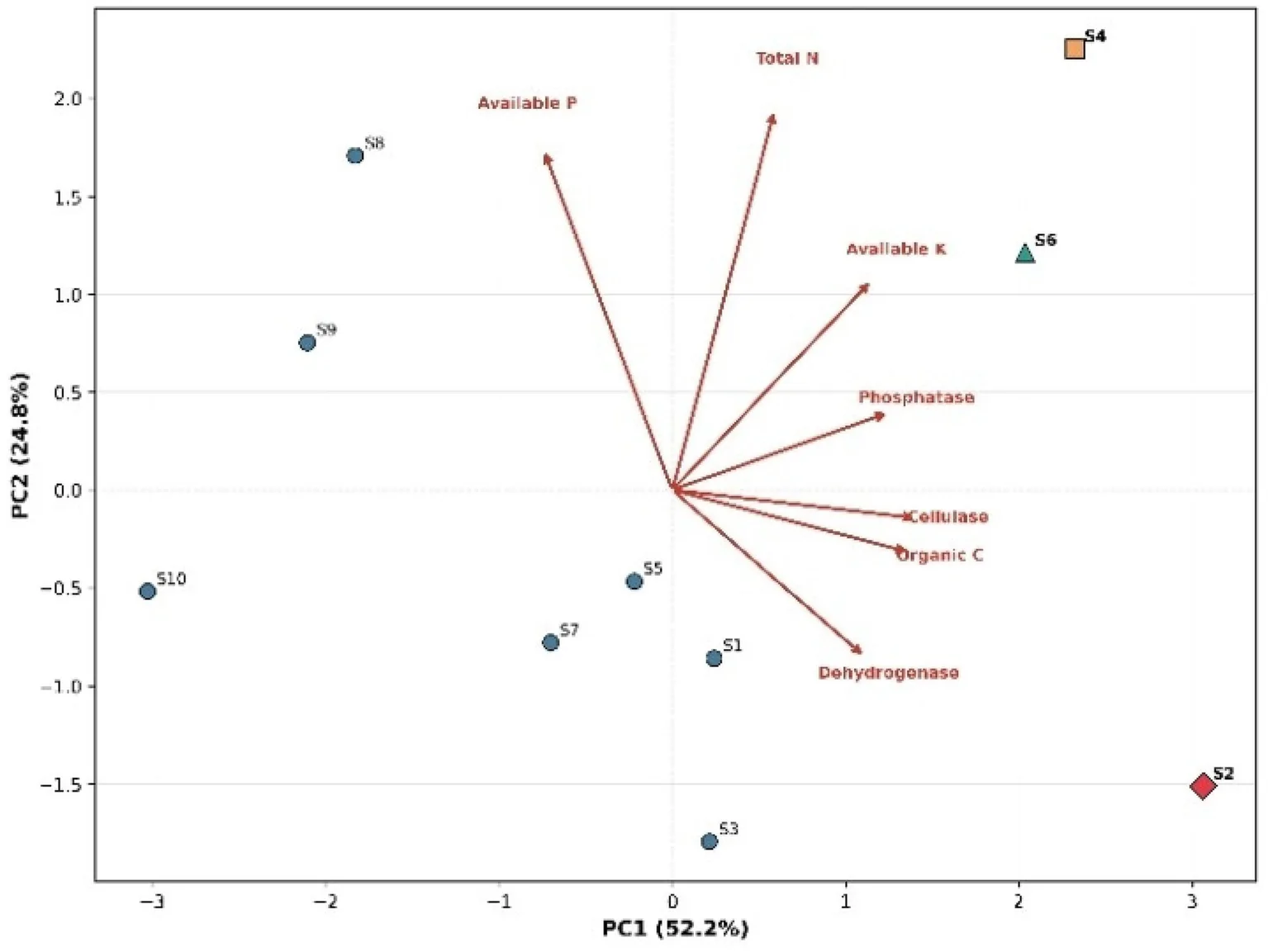
PCA Biplot- soil samples versus soil chemical and biological properties.

The chemical properties such as Total N, Available P and K and the biological properties such as cellulase, phosphatase and dehydrogenase activities were assessed for the 10 soil samples (S1 to S10). The results showed that the soil samples S1, S2, S3, S4 and S6 were positively correlated with the soil parameters Organic C, Total N, Available K, and the soil enzyme activities viz., cellulase, phosphatase and cellulase activities (Figure 2). While, the soil samples S5, S7, S8, S9 and S10 were negatively correlated. Among the positively correlated soil samples, the intensity of correlation was higher in the soil samples S2, S4 and S6. Therefore, these three soil samples were taken further for metagenomic studies. In metagenomic studies, the soil samples S2, S4, and S6 marked as Sample 1, Sample 2 and Sample 3, respectively.

### 3.2 Metagenomic sequencing

#### 3.2.1 Assembly of the metagenomes

A total of 105,872,212 sequencing reads were obtained from three soil samples. The data analysis encompassed a total of (38,373,652) reads in Sample-1, which consists of the deepest sequencing coverage compared to the other soil samples (31,986,874) & (35,511,686) reads in 2 and 3, respectively. Sample-3 showed the highest number of predicted genes (284,569), the largest total gene size (120,809,802 bp), and the longest average gene length (424.5 bp), as well as the longest maximum gene length (13,311 bp). These findings suggest that Sample 3 has a more complex function and a gene-rich microbial community compared to Samples 1 and 2, respectively. To initiate high-quality *de novo* assembly, raw sequencing reads were pre-processed to remove specific sequencing adaptors and low-quality reads (QV < 20). Metagenome assembly of the clean reads was performed using MEGAHIT v1.2.9, which is optimized for analysing large and complex data. The statistical elements of the metagenomic assemblies were calculated by using Perl scripts. Bacterial distribution across samples indicates that the majority of taxonomic diversity was captured in each sample.

#### 3.2.2 Taxonomic comparison of soil microbiome

**Figure 3:**
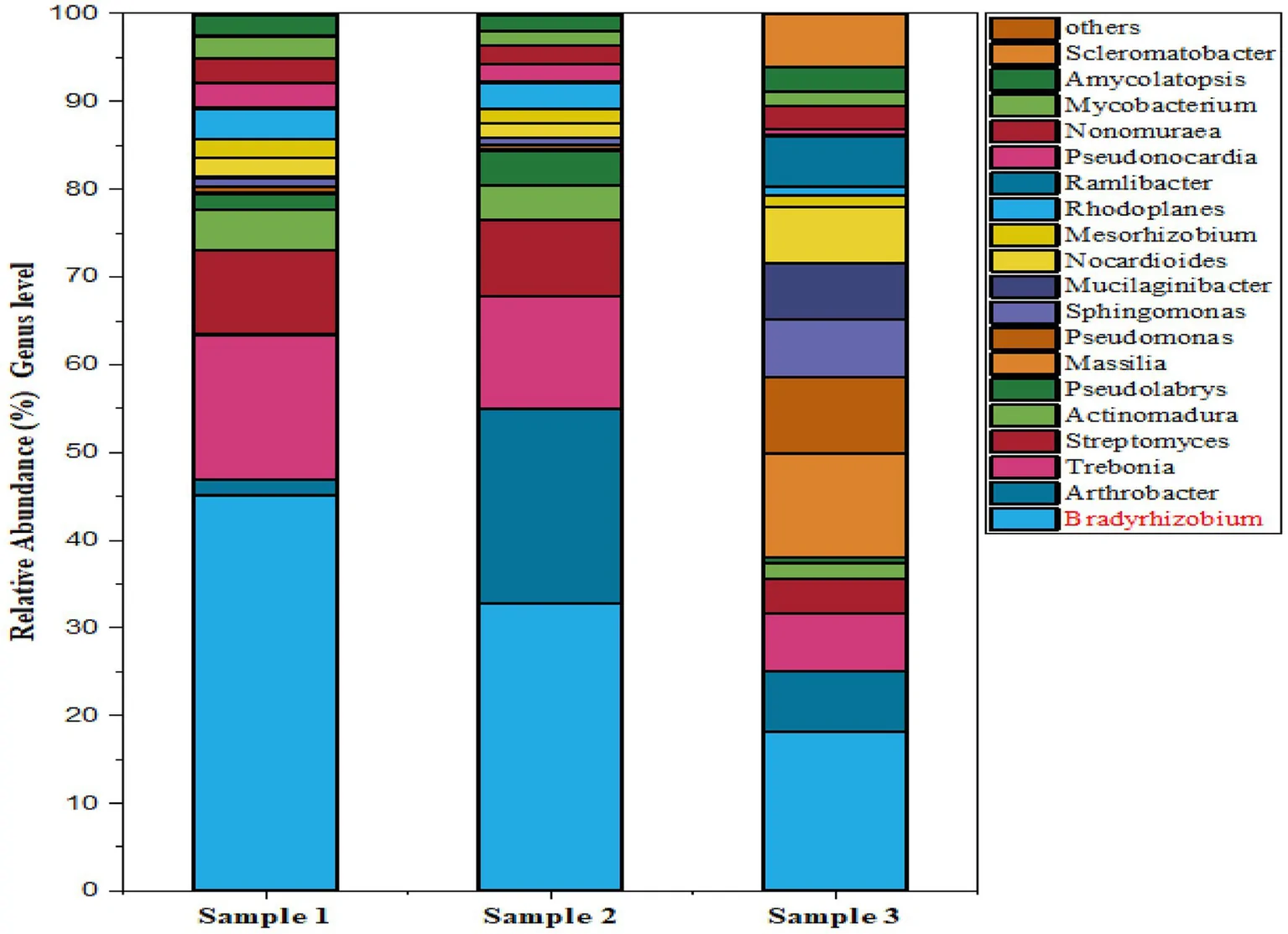
Relative abundance (%) genus level.

**Figure 4:**
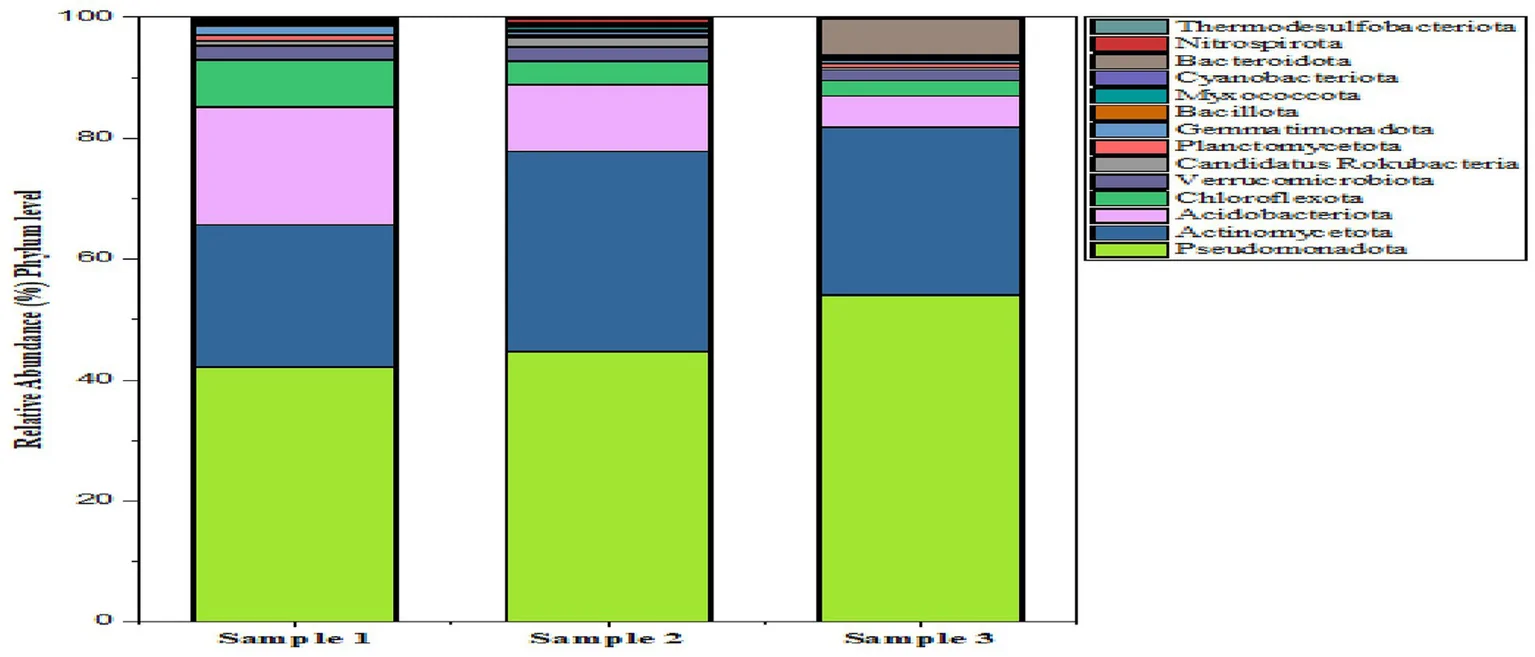
Relative abundance (%) phylum level.

Relative abundance of bacteria at genera and phylum level are shown in Figures 3, 4, respectively. An abundance of genus-level comparisons revealed notable differences in the microbial community structure among the soil samples. In sample 1, the dominance of *Bradyrhizobium* suggests its superficial and ecological role in nitrogen fixation and its adaptability to diverse soil conditions. *Trebonia* and *Streptomyces* are the next most abundant genera, followed by *Actinomonas*, which indicates a moderate diversity. However, the relative abundance patterns of subdominant taxa varied considerably. Conversely, sample 2 exhibited a greater abundance of *Arthrobacter* (10,563) and *Trebonia* (6,130) following *Bradyrhizobium*, suggesting a stronger representation of Actinobacteria and potentially a more metabolically versatile community, as represented in Figure 3. In sample 3, *Bradyrhizobium* remained the dominant genus (13,376), followed by *Mesorhizobium* (10,970), *Pseudomonas* (8,034), and *Massilia* (6,479). This distribution pattern indicates a more even taxonomic spread compared to the first two samples. Certain genera, such as *Micrococcus, Flavobacterium*, and *Sphingobacterium*, exhibited a higher relative abundance in sample 3, suggesting niche specialization and functional redundancy among the microbial groups. However, the relative abundance patterns of genus groups for subdominant taxa varied considerably. This shift highlights the potential differences in microbial community structure, which may be influenced by environmental conditions and plant-microbe interactions. The observed variation not only reflects microbial adaptability but also provides insights into potential functional diversity, which could be crucial for understanding ecosystem stability, resilience, and other applications. This even distribution reflects higher microbial diversity and functional potential.

Overall, the comparative assessment demonstrates that while *Bradyrhizobium* is a core genus across all three samples, the relative abundances of secondary taxa vary substantially. Sample 1 exhibits a dominance-dependent community with lower diversity. Sample 2 shows increased actinobacterial representation, and sample 3 displays the highest overall evenness and richness, indicating a more ecologically stable and functionally diverse microbial assemblage. While *Bradyrhizobium* remained a core genus across all samples, Sample 3 demonstrated the highest taxonomic evenness and diversity, suggesting a more ecologically stable and metabolically versatile microbial community compared to the other two samples.

Based on kaiju database analysis, the dominant bacterial orders identified in the samples were Burkholderiales, Hyphomicrobiales, Micrococcales, Streptosporangiales, Kitasatosporales, Mycobacteriales, Micromonosporales, Nostocales, Sphingomonadales, Pseudomonadales, and Propionibacteriales. The dominant bacterial phyla identified in the comparative samples were Pseudomonadota, Actinomycota, Acidobacteriota, Chloroflexota, Verrucomicrobiota, Bacillota, Myxococcota, Cyanobacteriota, Bacteroidota, and Nitrospirota (Figure 4).

#### 3.2.3 Krona chart

**Figure 5:**
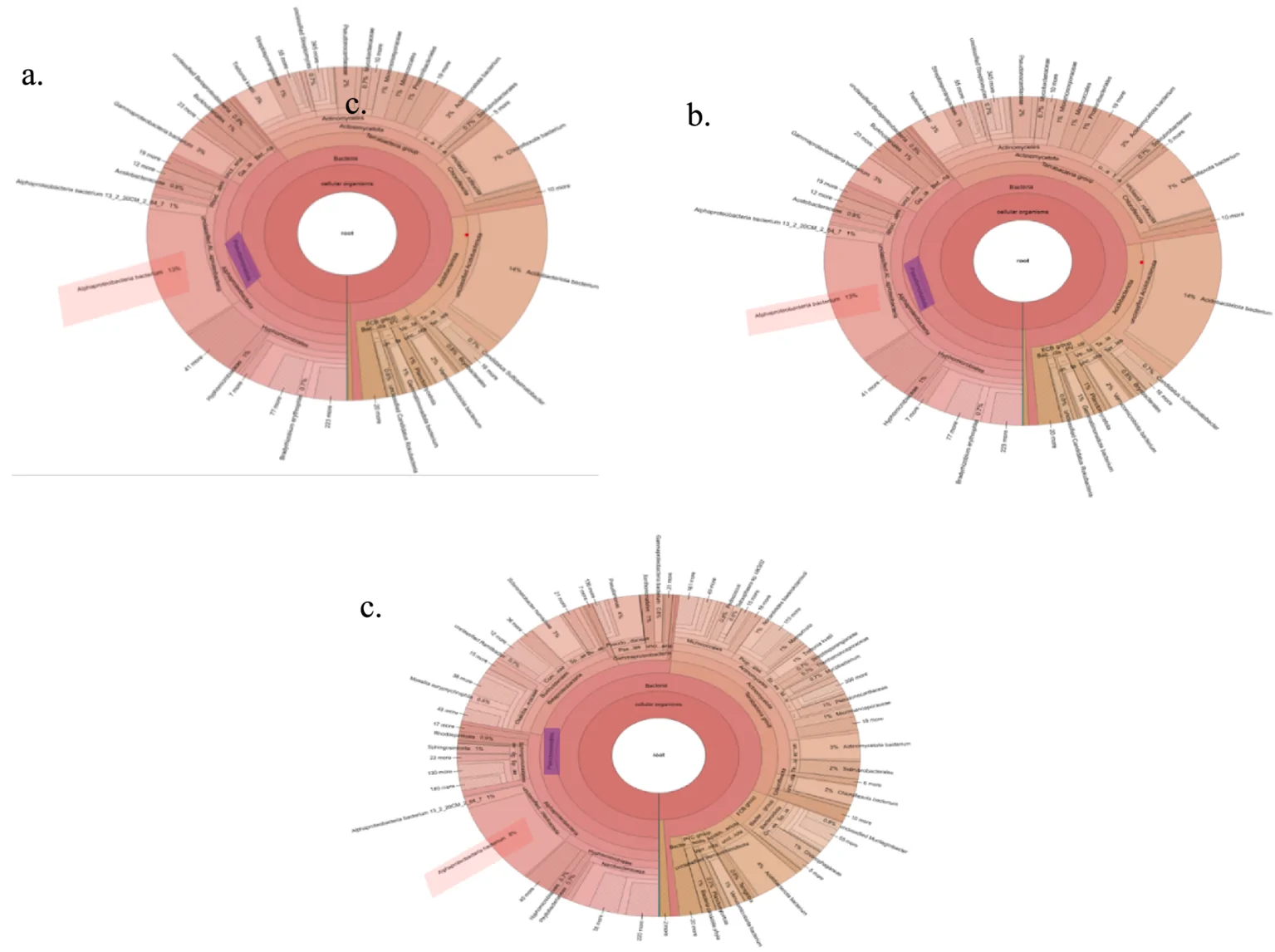
Krona chart images of **(a)** Nilgiris, **(b)** Coimbatore, and **(c)** Dindigul soil samples.

Comparative analysis of the three-krona charts revealed that the taxonomic profiles share a similar set of core root-associated microbes among the three soil samples (Figures 5a–c). Bacteria dominated in all the samples with distinct differences in relative abundance patterns, taxonomic richness, and the contribution of other minor groups. Among the bacteria, the class Proteobacteria stand out in every profile, which are the main players when it comes to colonizing roots and shaping the rhizosphere.

In the first profile, Alpha proteobacteria followed by the other classes while in the second and third profiles, Alpha, Gamma, and Beta proteobacteria all have a solid presence, which points the diversity in which these community’s work. *Acidobacteriota* comes in second for abundance across all three profiles. Interestingly, their numbers increased from the first to the third profiles. That uptick hints at more oligotrophic, soil-adapted taxa showing up in the later samples. Actinomycetota are around in all three, but they really take off in the third profile, both in terms of how many there are and how many different lineages show up. This suggests the third sample contains more decomposers and microbes that can produce secondary metabolites.

Likewise smaller groups like Chloroflexi, Bacteroidota, Gemmatimonadota, Firmicutes (specifically the Bacilli group), and Myxococcota spreaded out across all the profiles. Their numbers and diversity grow from the first to the third profile, which just shows the communities getting more complex. Archaea was only observed in the second profile. Most of them link back to the TACK group and Nitrososphaeria, depicting ammonia-oxidizing and boosting nitrogen cycling in that sample. At the finer taxonomic levels, the third profile stands out with the most low-abundance taxa and unclassified groups. Therefore, profile 3 had the richest and most even spread of microbes out of the three.

#### 3.2.4 Functional pathway annotation & PGP attributes

**Figure 6:**
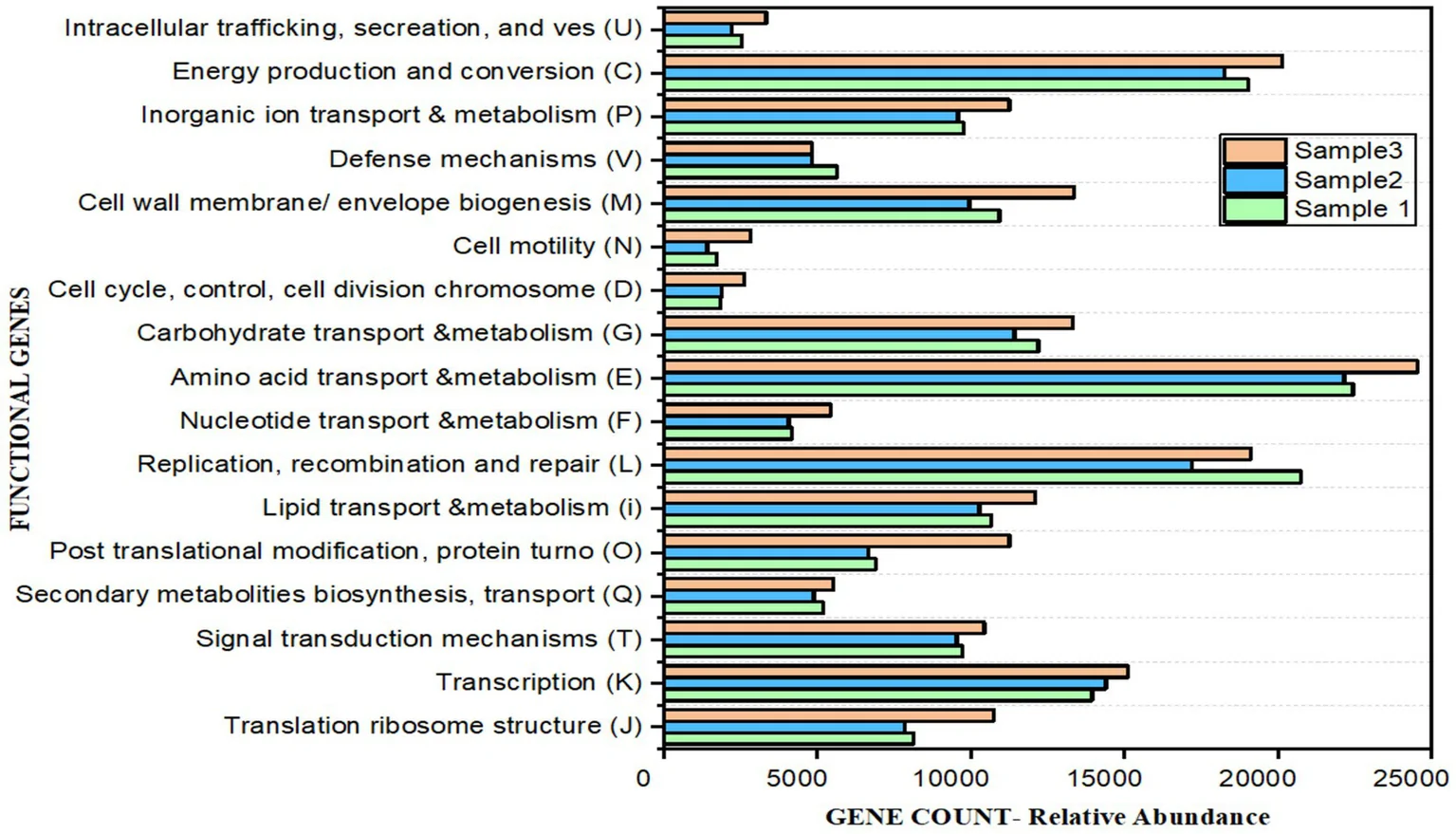
Functional abundance of soil metagenomes. The coloured bars represent the functional values in Nilgiris soil (olive green), Coimbatore soil (sky blue), and Dindigul soil (pale orange).

The metagenomic functional comparison mapped the abundance profile of different functional categories across the three soil samples in the Western Ghats. The functional category of the soil samples was identified by the abundance of functional reads mapped to the highest distribution levels and categorized similarly across all three soil samples. Metagenomic functional analysis of soil samples was performed using COG databases (Figure 6). In these functions, most of the reads were highly distributed within the pathways of amino acid transport and metabolism (E), Replication, recombination and repair (L), Energy production and conversion ©, Carbohydrate transport and metabolism (G), Lipid transport & metabolism (I), and Inorganic ion transport & metabolism (P). Metagenomic analysis revealed a treasure trove of bioactive secondary metabolites and their plant growth-promoting bacteria in Western Ghats soils. Each functional level’s hit counts were summed, and the percentages of hits for each level relative to the total number were compared (Figure 6).

Comparative functional groups show an enrichment of key functional groups important for plant and microbial communities. Genes involved in energy production have higher abundance, indicating greater metabolic efficiency that supports nutrient cycling and organic matter decomposition. The dominance of carbohydrate and amino acid transport and metabolism suggest greater carbon and nitrogen availability, which in turn boosts root formation, biomass production, and soil fertility. The functional abundance of translation, transcription, and replication, recombination, and repair genes indicate active microbial growth, genomic stability, and adaptability. Inorganic ion transport and metabolism enhance the potential for greater mobilization of phosphorus, iron, and micronutrients. In addition, the dominance of defense mechanisms, post-translational modification, and protein turnover genes suggests better stress tolerance and functional efficiency. The higher abundance of secondary metabolite biosynthesis genes robustly enhances the production of antibiotics, siderophores, and other secondary metabolites that inhibit phytopathogens and support plant defense. Finally, the enrichment of genes related to cell wall/membrane biogenesis, cell motility, and intracellular trafficking and secretion suggests better rhizosphere efficiency and effective root colonization.

Likewise, the third profile, Dindigul soil, has a multifunctional abundance of functional genes and facilitates strong plant growth-promoting and nutrient cycling microbial community. And, it contains more decomposers and microbes capable of producing secondary metabolites. Therefore, profile 3 had the richest and most even distribution of microbes among the three samples.

#### 3.2.5 Alpha diversity

**Figure 7:**
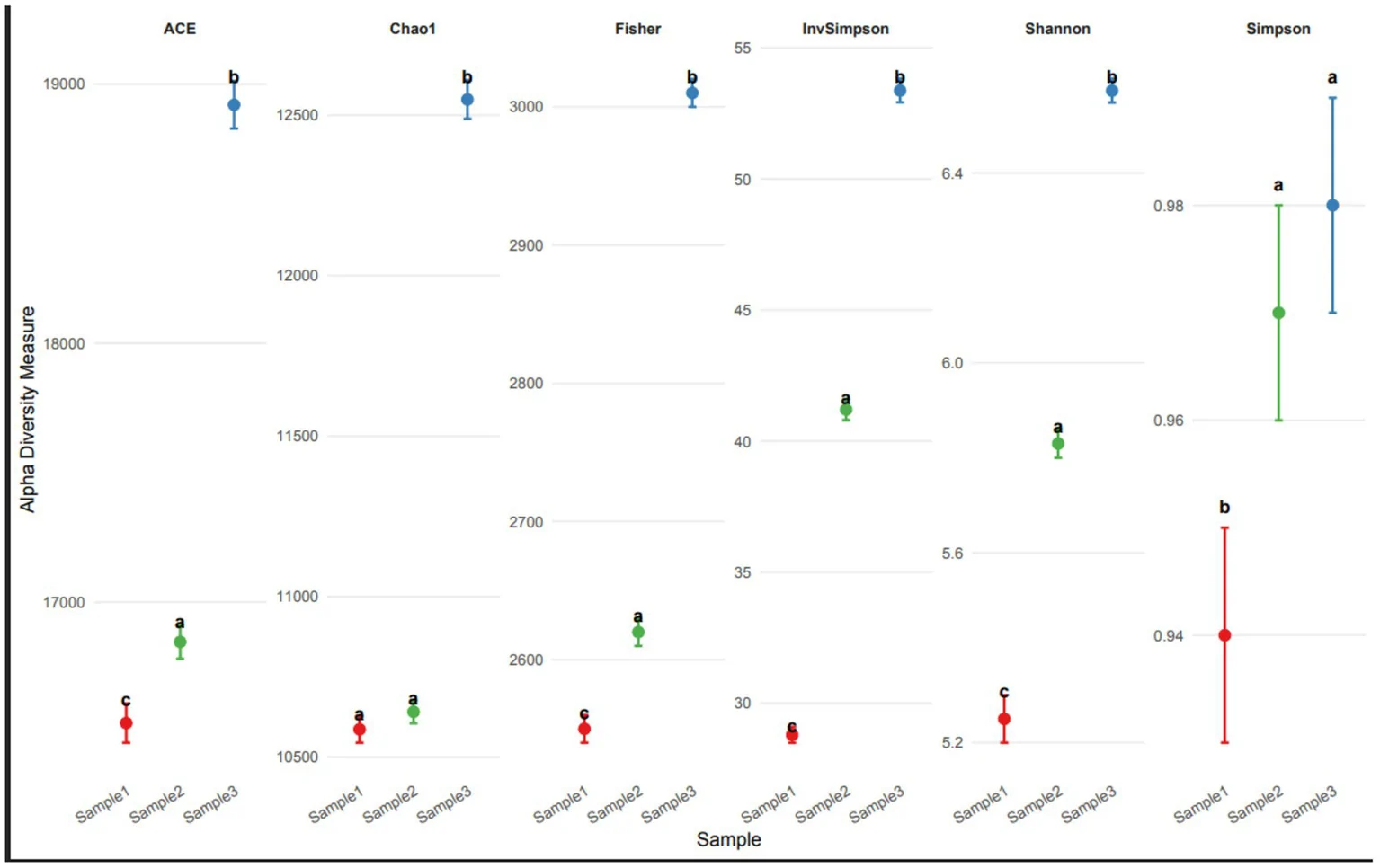
Sample-wise alpha diversity indices, determined by QIIME analysis (Chao1, Shannon, Simpson, ACE, Inv Simpson, and Fisher), are represented as mean ± SD (*n* = 3). Different letters indicate significant differences among samples based on one-way ANOVA followed by Tukey’s HSD test (*p* < 0.05).

Alpha diversity analysis revealed remarkable variation in microbial richness, diversity, and community heterogeneity among the analyzed samples (Figure 7). Richness estimators, such as Chao1 and ACE, provided evidence of a high degree of taxonomic complexity and structural diversity within the microbial communities. Similarly, diversity indices such as Shannon, Simpson, and Fisher’s alpha indicated elevated species evenness and a well-balanced distribution of taxa. The species richness and evenness of sample 3, suggest that the existence of an ecologically diverse and heterogeneous microbial community in the studied environment.

#### 3.2.6 Beta diversity

**Figure 8:**
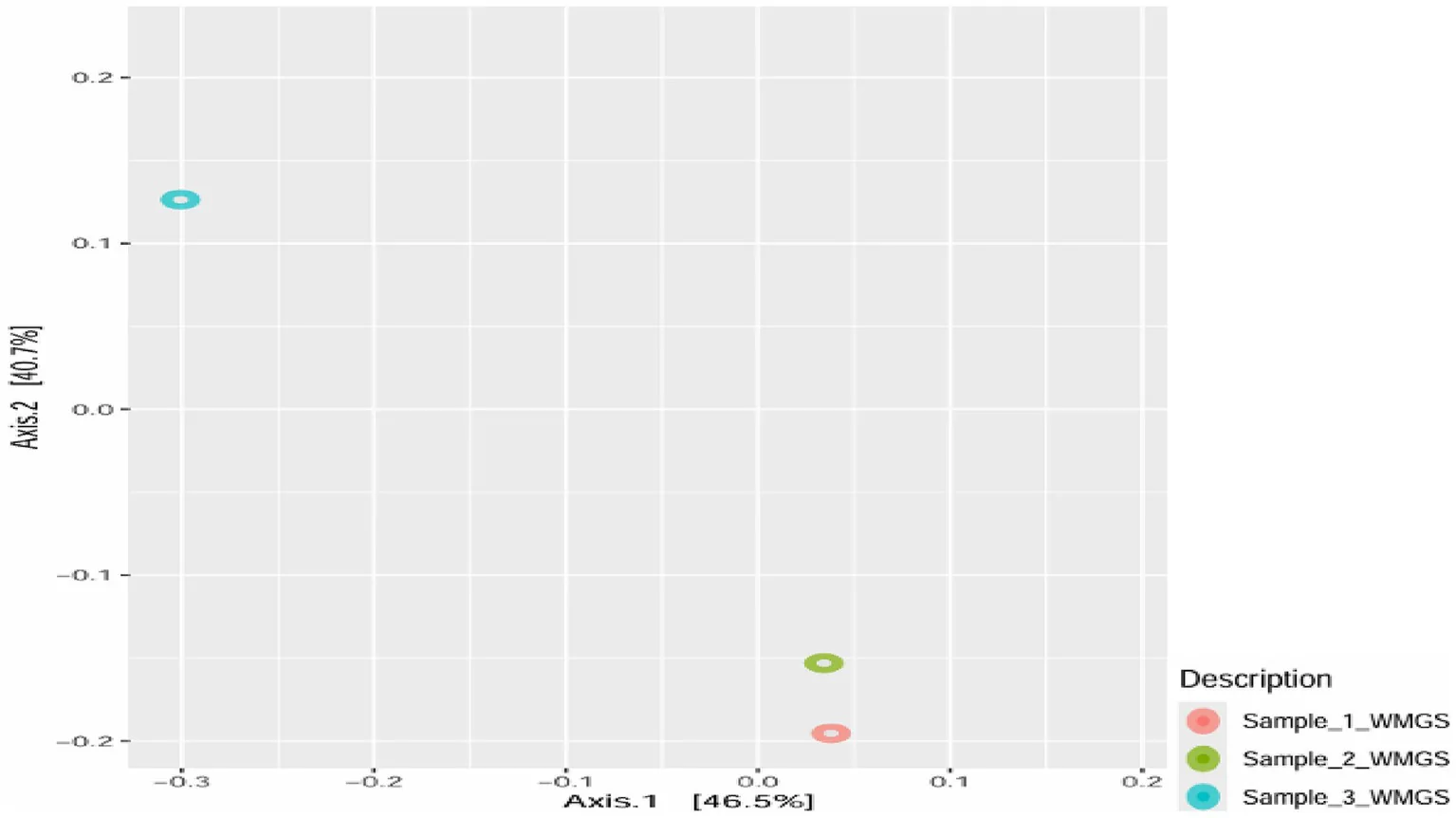
Principal coordinates analysis (PCoA) plot.

Comparing the beta diversity revealed clear compositional differences in microbial community structure among the analyzed samples (Figure 8). The first principal coordinate, Axis 1, explained 46.5% of the total variation, while the second coordinate, Axis 2, accounted for an additional 40.7%, together capturing a substantial proportion of the overall community dissimilarity. In contrast, Samples 1 and 2 clustered closer together along Axis 1 but were separated along Axis 2, suggesting partial similarity in community composition, with detectable differences in relative taxonomic abundance patterns. The dispersion of points across both axes reflects heterogeneity among samples, with Axis 1 primarily driving the major compositional shift and Axis 2 contributing to secondary variation. Overall, the PCoA results demonstrate pronounced beta diversity among the samples, highlighting distinct microbial community structures, particularly for Sample 3. In contrast, Samples 1 and 2 exhibit comparatively closer but still distinguishable community profiles.

### 3.3 Isolation and enumeration of bacteria

The bacterial populations were enumerated, and the mean value (average) is represented in CFU/g of soil, as indicated in Table 2. The statistical results showed that the bacterial population in the studied soil samples ranged from 81 to 131 (10^6^ CFU/g of soil). The highest and lowest population was recorded in S6 and S10, respectively.

**Table 2:** Colony-forming unit (CFU/g soil) of bacterial populations.

Soil samples	10^6^ CFU/g of soil
S1	124.00 ± 7.211^b^
S2	82.000 ± 7.000^i^
S3	96.333 ± 8.504^g^
S4	96.666 ± 8.736^f^
S5	92.333 ± 5.131^h^
S6	131.66 ± 6.506^a^
S7	112.66 ± 5.686^c^
S8	106.66 ± 8.504^d^
S9	97.66 ± 7.023^e^
S10	81.667 ± 7.023^j^

S, denotes soil samples. Values are means of triplicate determination (*n* = 3) ± standard deviation (SD), statistically significant *p* < 0.05, where a > j in the column.

### 3.4 Plant growth-promoting (PGP) traits characterization

**Figure 9:**
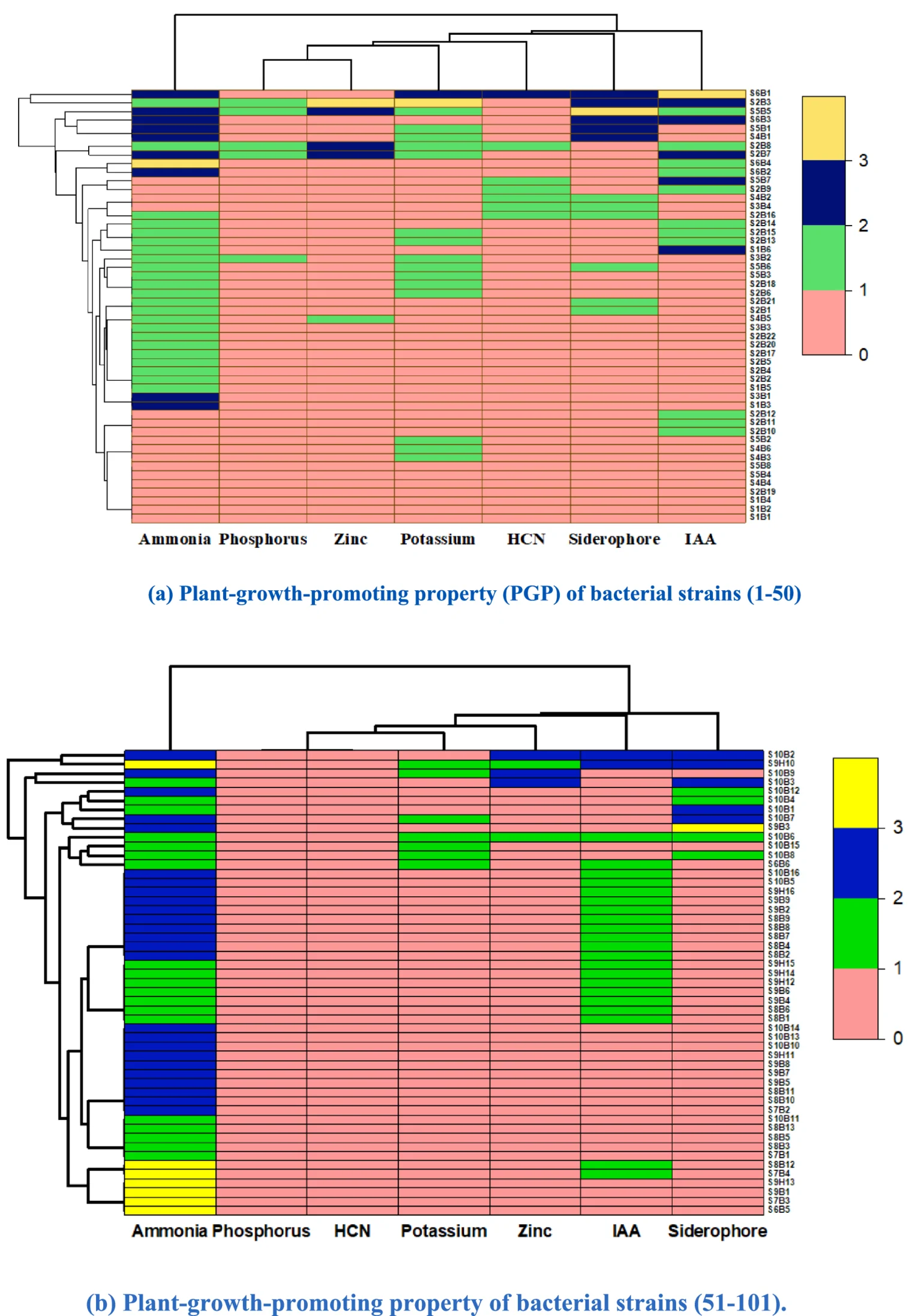
Plant growth promoting properties (PGP) of bacterial isolates. 1–50 **(a)** and 51 to 101 **(b)**. The scoring was based on the following four categories: Absent (0), Low (1), Medium (2), High (3) corresponding to pale pink, green, blue, and yellow in colour, respectively.

One hundred and one bacterial isolates were screened for variable PGPR qualitative traits characterization and plotted using a heat map method (Origin Pro 2025 software). Plant growth promotion (PGP) characteristics, such as Ammonia production, Phosphate, Potassium, Zinc solubilization, Siderophores, Indole acetic acid (IAA), HCN, and Nitrogen fixation property, were screened. Details are presented in Figure 9. The plant growth-promoting property of bacterial strains (1–50 nos) is given in Figure 9a. While, 51 to 101 is shown in Figure 9b. Among the various properties, ammonia production has been detected at its maximum, while HCN and phosphorus solubilization have been detected at their minimum. The plant growth-promoting property has been variedly distributed among the bacterial isolates.

The culturable bacterial isolates were screened based on a qualitative PGP traits composite scoring system depends on the criteria of absent, low, medium and high and the corresponding values of 0, 1, 2, and 3, respectively. Based on the overall scores obtained from all the PGP characters, the first four best-performing bacterial isolates (S2B3, S10B2, S6B1, S9H10) were selected, and their respective scores were 12, 8, 11, and 9 (Supplementary Table 1). Out of the 101 bacterial isolates, 43 bacterial isolates were found to be gram-negative, and 67 bacterial isolates were gram-positive (data not shown).

### 3.5 Characterization of the selected bacterial isolates

The morphological and biochemical characterization of the selected isolates (S2B3, S10B2, S6B1, S9H10) is given in Supplementary Tables 2, 3, respectively. The quantitative determination of potassium solubilization, IAA production, and percent inhibition against fungal plant pathogens of the selected four bacterial isolates is given in Table 3. The morphological and biochemical characterization revealed that the isolates are Gram-negative, rod-shaped, and translucent, belonging to the *Pseudomonas* sp. The potassium solubilization (μg/ml) was recorded in the range of 0.41 to 1.93, with the highest rate of solubilization (1.93) observed in isolate S2B3. While the highest IAA production (μg/ml) of 15.76 was observed in the isolate S6B1, the range of IAA production was recorded as 0.22 to 15.76. The isolate, S10B2, showed a wide spectrum of inhibition against *Rhizoctonia* solani (81.25%), *Sclerotium rolfsii* (70.1%), and *Fusarium oxysporum* (35%). The isolate S2B3 shows moderate inhibitory concentration with 13.75 and 15% inhibition towards *R. solani* and *F. oxysporum,* respectively. But there is no inhibition towards *S. rolfsii.* The isolate S9H10 shows no antagonistic activity, while S6B1 inhibits 5% of *F. oxysporum* but exhibits no inhibition against the other fungal pathogens.

**Table 3:** Quantitative determination of plant growth promotion property of selected bacterial isolates.

Bacterial isolates	Potassium (μg/ ml) released	IAA (μg/ ml) released	% Inhibition		
			*Rhizotonia solani*	*Sclerotium rolfsii*	*Fusarium oxysporum*
S2B3	1.90 ± 0.92^a^	0.21 ± 0.165^d^	13.75	ND	15
S10B2	0.44 ± 0.13^d^	0.24 ± 0.115^c^	81.25	70.1	35
S6B1	1.54 ± 0.14^b^	15.3 ± 0.455^a^	ND	ND	5
S9H10	0.56 ± 0.80 ^c^	0.37 ± 0.142^b^	ND	ND	ND

Values are means of triplicate determination (*n* = 3) ± Standard deviation is statistically significant, *p* < 0.05, where a > b > c > d in each column.

### 3.6 Molecular characterization

**Figure 10:**
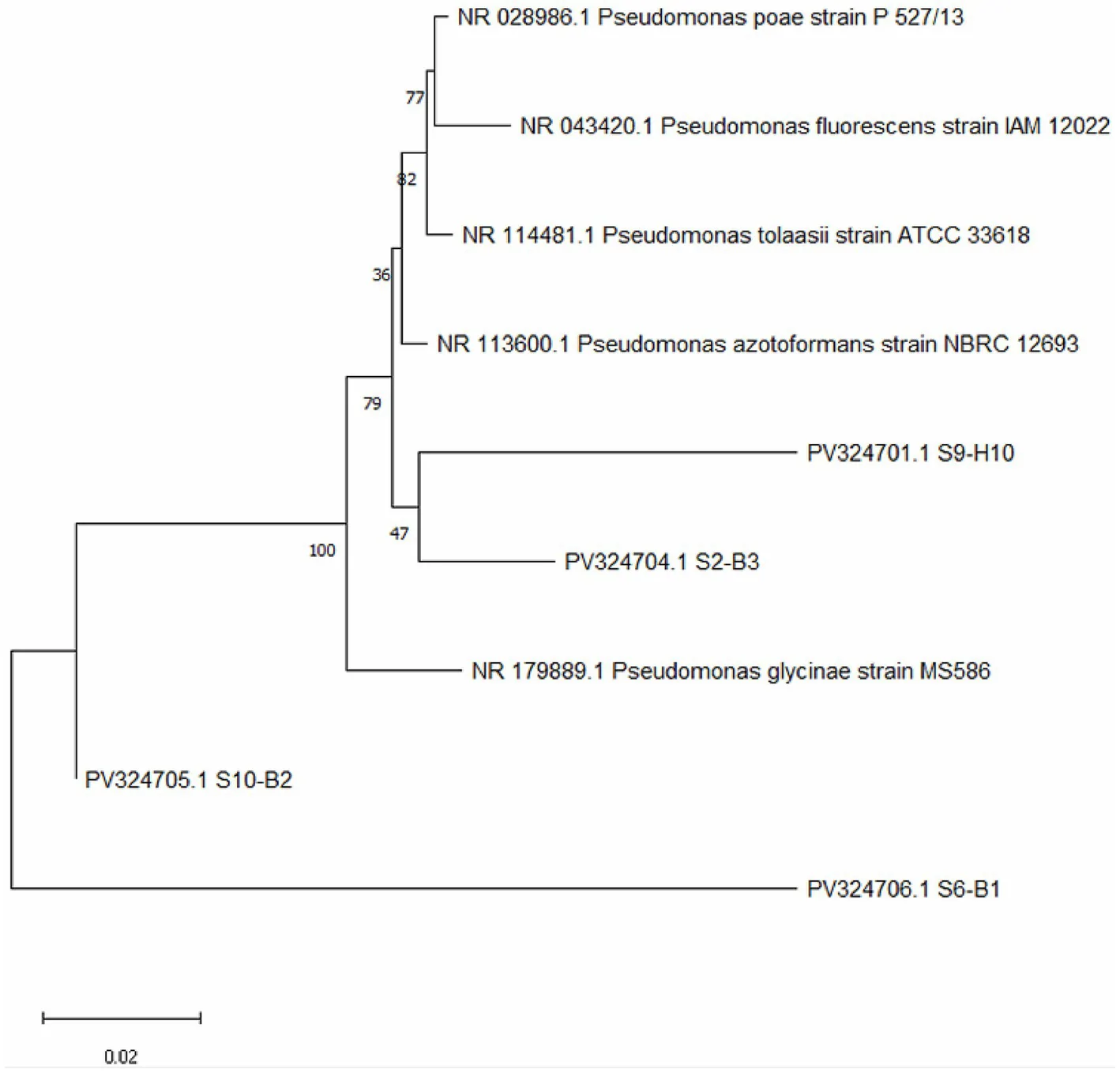
Phylogram of the selected bacterial isolates.

The 16 s rDNA analysis revealed the bacterial isolates were *Pseudomonas glycinae* (S6B1), *Pseudomonas tolaasii* (S2B3), *Pseudomonas azotoformans* (S9H10), and *Pseudomonas poae* (S10B2). The 16S rRNA sequences of the above-mentioned four isolates, as analysed by BLAST in NCBI, are presented in a phylogenetic tree format (Figure 10). The bacterial sequences were submitted to NCBI under the accession numbers: PV324701 (S9H10), PV324704 (*S2B3*), PV324705 (*S10B2*), and PV324706 (*S6B1*).

### 3.7 Evaluation of selected bacterial isolates on the growth of chickpea

The summary of the results of the effect of bacterial isolates on root and shoot length and fresh and dry biomass in chickpea under agar plate, paper-towel and pot culture conditions are presented in Table 4.

**Table 4:** Effect of bacterial isolates on the growth of chickpea in agar plate, paper towel, and pot culture experiments.

Bacterial isolates	Root length (cm)	Shoot length (cm)	Fresh biomass (g)	Dry biomass (g)
Agar plate				
Control	9.68 ± 3.305^b^	2.14 ± 0.472^b^	0.73 ± 0.110^b^	0.14 ± 0.237^b^
S9H10	13.84 3.074^a,b^	2.74 ± 0.466^a,b^	0.85 ± 0.067^a,b^	0.15 ± 0.027^b^
S6B1	9.50 ± 1.870^b^	3.10 ± 0.959^a^	0.89 ± 0.110^b^	0.22 ± 0.042^a^
S10B2	14.58 ± 3.95^a^	2.92 ± 1.742^a^	0.82 ± 0.124^b^	0.18 ± 0.363^a,b^
S2B3	14.30 ± 4.816^a^	2.04 ± 1.810^a^	0.82 ± 0.107^b^	0.15 ± 0.017^b^
Paper towel				
Control	19.00 ± 7.834^b^	17.90 ± 3.228^b^	0.92 ± 0.186^b^	0.16 ± 0.022^a^
S9H10	19.74 ± 4.202^b^	18.30 ± 6.068^b^	0.96 ± 0.055^b^	0.18 ± 0.048^a^
S6B1	21.00 ± 0.790^a,b^	21.50 ± 5.044^a^	1.08 ± 0.213^a,b^	0.13 ± 0.019^b^
S10B2	23.20 ± 2.280^a^	21.20 ± 3.327^a^	1.09 ± 0.042^a,b^	0.16 ± 0.025^a^
S2B3	23.58 ± 1.953^a^	19.84 ± 4.878^a,b^	1.15 ± 0.156^b^	0.16 ± 0.025^a^
Pot culture				
Control	5.73 ± 1.069^b^	13.50 ± 2.291^b^	1.53 ± 0.102^b^	0.33 ± 0.018^b^
S9H10	8.87 ± 2.136^a^	18.76 ± 1.965^a^	1.88 ± 0.276^b^	0.34 ± 0.026^b^
S6B1	9.00 ± 2.291^a^	15.20 ± 1.311^ab^	1.88 ± 0.276^b^	0.39 ± 0.064^a^
S10B2	8.30 ± 1.374^a^	13.73 ± 1.750^b^	1.69 ± 0.174^a,b^	0.39 ± 0.049^a^
S2B3	8.50 ± 1.044^a^	13.20 ± 0.400^b^	1.57 ± 0.137^b^	0.35 ± 0.028^b^

Values are means of quintuplicate determination (*n* = 5) ± Standard deviation for the Agar plate, Paper Towel, and values are means of triplicate determination (*n* = 3) ± Standard deviation for the Pot culture technique. The treatment with the same superscript within a column does not differ significantly at *p* = 0.05.

#### 3.7.1 Agar plate method

The findings reveal that the growth parameters of chick pea plant (root length, shoot length, fresh and dry biomass) exhibited an increase in PGP-treated chickpea plants when compared with the uninoculated control. The highest root length (>14 cm) was recorded in in S10B2 and S2B3 treatments. While, the shoot length, fresh and dry biomass were higher in the chickpea seeds treated with the isolate, S6B1, compared to the control.

#### 3.7.2 Paper–towel method

The application of PGP isolates was found to significantly impact most of the growth parameters of chickpea plants compared with the uninoculated control in the paper towel method. The root length was higher (>23 cm) in S10B2 and S2B3 treatments, compared to the control. The shoot length was higher in S6B1 and S10B2 treatments (>21 cm), compared to the control. The fresh and dry biomass was higher in S2B3 and S9H10 treatments, respectively.

#### 3.7.3 Pot culture study

In the pot culture assay, inoculating chickpea with PGPR isolates resulted significant improvements in growth factors compared to the uninoculated control. Among the various treatments, the bacterial isolates S6B1 and S9H10 were the most effective in elongating the roots, with each achieving a root length of 9 cm, while the control recorded a mere 5.73 cm. Similarly, shoot length experienced a significant increase in S9H10-inoculated plants, with the highest shoot length of 18.76 cm, followed by S6B1 (15.20 cm), compared to the control (13.50 cm). Fresh biomass accumulation was recorded higher in S6B1 and S9H10 treatments, 1.88 g compared to the control (1.53 g). In dry biomass, S6B1 and S10B2 showed the highest value, 0.39 g, in comparison to the control (0.33 g).

## 4 Discussion

At the sampling sites, the soil texture was predominantly silt, with a transition from laterite red loam to reddish-brown, and a pH range of 4.0 to 7.0. The temperature ranged from 7 °C to 25 °C. Acidic environments are characterized by enhanced soil moisture, frequent rainfall, humus accumulation, and cool temperatures. This region is generally renowned for its cultivation of plantation crops, temperate vegetables, flowers, and fruits that flourish in these agro-climatic conditions, which are well-suited to acidic soils (Pathak et al., 2021).

Whole metagenomic sequencing reveals the taxonomic analysis of microbial families, orders, and genera associated with the soil samples (Iqbal, 2026). Microbial community composition and relative abundance were influenced by local environmental conditions and soil properties. Metagenomic sequencing also explores the taxonomic classification and functional pathway of the microbial communities present in the soil samples. Metagenomics primarily relies on sequencing to create genetic libraries. In this context, bioinformatic data can reconstruct microbial metabolism, thereby predicting functional pathways in soil ecosystems. Comparative metagenomic analysis of all three samples revealed notable differences in environmental conditions, microbial community structure, sequencing depth, gene properties, and functional diversity.

The results showed sample 1 has deeper sequencing coverage, its relatively low gene diversity and abundance may indicate a less intricate microbial community. While *Bradyrhizobium* was the core genus, the relative abundance of subdominant taxa showed great variation among the samples. *Trebonia, Streptomyces*, and *Actinomonas* co-existed with *Bradyrhizobium* in sample 1, indicating a moderate level of diversity within the community. The relative abundances in the Sample 2 included *Streptomyces*, *Actinomonas*, *Pseudolabrys*, and *Pseudomonas* highlighting the enrichment of genera associated with organic matter decomposition and antibiotic production. The prevalence of *Arthrobacter* and *Trebonia* suggests a swift transition towards an actinobacteria-dominated community with more metabolic adaptability. The prevalence of *Streptomyces* and members from the Actinobacteria phylum in sample 2 and 3 underscores their role in soil fertility and the synthesis of antibiotics, functions that are highly relevant for nutrient cycling and microbial competition (Wankhade et al., 2025; Rajan et al., 2026). Actinobacteria are recognized for their ability to thrive in fluctuating environmental conditions and make a significant contribution to soil carbon turnover (Kurtboke, 2017).

The sample 3 was taxonomically distributed, dominated by *Bradyrhizobium, Mesorhizobium, Pseudomonas*, and *Massilia*. The increased relative abundance of *Mesorhizobium* and *Pseudomonas* suggests a community with a higher capacity for nitrogen fixation and greater functional flexibility. These genera are associated with plant symbiosis, denitrification, and degradation of complex organic compounds (Mankoti et al., 2024). The nitrogen-fixing and plant growth-promoting bacteria, are known to colonize various parts of plant tissues (Barka et al., 2016). Moreover, the presence of Micrococcus, Flavobacterium, and Sphingobacterium indicates niche specialization and functional redundancy, characteristics frequently observed that enhance ecosystem resilience. The findings of the study suggest that Sample 3 has a more functionally diverse and metabolically complex microbial population.

Among the identified genera, *Bradyrhizobium* was found in all three soil samples. The presence of *Bradyrhizobium* was detected in various soil samples, indicating its robust ecological role in Indian agroecosystems (Kumar et al., 2023). *Bradyrhizobium* is widely recognized for its symbiotic nitrogen-fixing ability, modulation of plant signaling pathways, oxidative stress mitigation, and its resilience in acidic and nutrient-poor soils, thereby contributing to its functional roles in soil fertility and plant productivity (Delamuta et al., 2013; Khan et al., 2024; Tharanath et al., 2024). The dominance of *Bradyrhizobium* strains obtained from soybean cultivated in Indian acidic soils was evident in genetic aspects and functional richness diversity (Appunu et al., 2015). The higher representation of *Mesorhizobium* and *Pseudomonas* suggests an enhanced nitrogen fixation potential and metabolic versatility, as both genera are well-known for their roles in plant symbiosis and growth promotion (Chandran et al., 2021; Hnini and Aurag, 2024).

Overall, the comparative analysis revealed that *Bradyrhizobium* is a core genus in acidic soil samples, while shifts in secondary taxa indicate differential ecological strategies and metabolic capacities well-suited to the acidic environment. Sample 1 with pH 5.05 is dominated by a single type and hence has lower diversity. Sample 2 with pH 7.2 is characterized by a stronger actinobacterial enrichment, while Sample 3 with pH 5.95 presents the highest evenness and taxonomic richness. In a similar study, the predominance of *Bradyrhizobium* was noticed in acidic soil of Central European Forest. Soil pH is the major driving force of soil bacterial community, diversity and function. The other environmental factors including nutrient availability, organic matter content, pH, and plant-microbe interactions (Kaiser et al., 2026). Due to adaptation to specific environmental conditions and nutrient levels, the existence of microorganisms with more extensive functional capabilities is indicated by larger gene sizes and counts. Collectively, the findings emphasize that community structure at the genus level not only mirrors ecological adaptation but also underpins the functional potential of soil microbiomes, which are crucial for biogeochemical cycling and the maintenance of sustainable ecosystem functioning.

**Figure 11:**
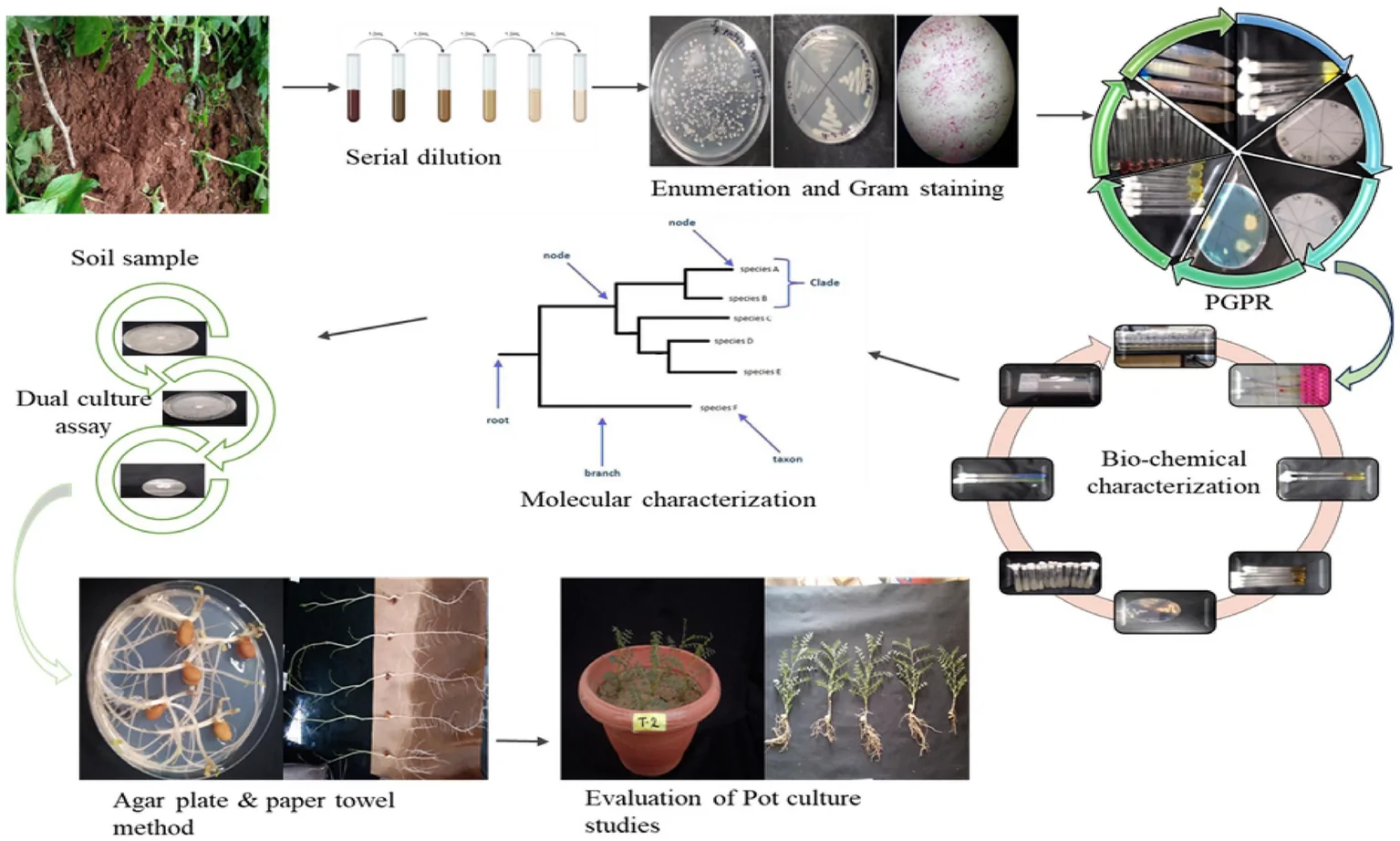
Overall schematic representation of the study of culturable bacteria.

In the culturable study, an attempt was made to isolate, screen, and characterize plant growth-promoting bacteria from soil samples. The overall schematic representation of the study of culturable bacteria is given in Figure 11. The soil samples were found to be rich in bacterial diversity (10^6^ CFU/g of soil), and 101 bacterial strains were isolated in this study. Out of the 101 bacteria, four isolates (S2B3, S10B2, S6B1, S9H10) showed outstanding results in qualitative screening of plant growth-promoting traits, viz., ammonia production, phosphate, potassium, and zinc solubilization, siderophore production, hydrogen cyanide (HCN), and IAA production. The selected bacterial isolates were characterized for morphological, biochemical, 16S rRNA sequencing, antagonistic property, potassium solubilization, and IAA production.

The morphological, biochemical, and 16S rRNA sequencing revealed that all the isolates belong to *Pseudomonas* sp. The phylogram illustrates the relatedness of the isolates based on BLAST analysis with the sequences of *Pseudomonas* sp. extracted from the NCBI database. The evolutionary history was inferred using the ClustalW (Thompson et al., 1994) and neighbor-joining method (Saitou and Nei, 1987). The results revealed the identity of the bacterial isolates as *Pseudomonas glycinae* (S6B1), *Pseudomonas tolaasii* (S2B3), *Pseudomonas azotoformans* (S9H10), and *Pseudomonas poae* (S10B2).

Interestingly, the present study showed the predominant genus in unculturable and culturable studies were *Bradyrhizobium* and *Pseduomonas*, respectively. In unculturable studies, the genus *Pseudomonas* occurred as associate taxa; however, they have encountered predominant under culturable conditions. These contrasting results might be due to versatile growth characteristics including faster growth rate of *Pseudomonas* compared to slow growing *Bradyrhizobium*. Numerous researchers have reported that the genus *Pseudomonas* sp. belongs to Proteobacteria and *Bacillus* sp. belongs to Firmicutes, contribute significantly to plant growth promotion. They are found in the rhizospheric soils and considered as an important PGPR (Fatemeh et al., 2014). Previous studies suggested that the Actinobacteriota and Proteobacteria were the most abundant rhizospheric phyla in all seasons (Mendes et al., 2011; Krstić Tomić et al., 2023). A high abundance of *Bacillus*, a genus rich in PGPB was observed at lower taxonomic levels (Narayanan et al., 2022). PGPR is considered as a safe, eco-friendly biotechnological approach. The application of *Pseudomonas* sp. in modern agriculture results in reduced rates and the elimination of chemical fertilizers (Duca et al., 2014).

In the dual culture assay, *P. poae* S*10B2* exhibited the maximum inhibition against all three fungal pathogens, which showed its biocontrol potential. The quantitative determination of PGP property showed, the strain *P. glycinae* S6B1 exhibited higher potassium solubilization and IAA production rate. In plant growth, indole-3-acetic acid, derived from auxin, plays a dynamic role, produced by bacteria and fungi. IAA initiates root and plant development, playing a crucial role in cell division and elongation during plant growth promotion (Phillips et al., 2011; Mohite, 2013). The effect of seed bacterization with selected bacterial isolates on chickpea plant growth parameters was evaluated in agar plate, paper towel, and pot culture experiments. The improved root and shoot length and fresh and dry biomass in chickpea plants were observed upon inoculation of seeds with *P. glycinae* S6B1. Photosynthesis is the primary metabolic process by which plants grow. PGPR enhances chlorophyll formation in plant leaves, thereby improving photosynthesis and biomass accumulation (Nadeem et al., 2010). The positive effect of inoculation of *Pseudomonas* sp. on the growth and yield of rice, radish, sorghum, and pearl millet has been reported (Raj et al., 2004). Thus, the results of the present study revealed that *P. glycinae* S6B1 possesses multifarious plant growth-promoting traits and triggers vegetative growth and biomass accumulation in chickpea, validating its potential as an effective bioinoculant in chickpea production.

## 5 Conclusion

The microbiome analysis revealed the novel insights of prevalence of Pseudomonodata in the soil samples studied. Though the genus *Bradyrhizobium* was prevalent in all the soil samples, the highest evenness and taxonomic richness was found in Dindigul soil sample. In culturable studies, a total of 101 bacterial isolates were obtained. Four bacterial isolates (S6B1, S2B3, S9H10 and S10B2) were identified as promising based on their plant growth-promoting characteristics. The bacterial strain *P. glycinae* S6B1 was found to possess multifaceted plant growth-promoting properties, and enhance the growth parameters of chickpea. By tailoring unique functional features, the elite strain *P. glycinae* S6B1 can be utilized as a bio-stimulant and as a potential growth-promoting agent. Future research may focus on development of synthetic microbial communities (SynComs) based on *Bradyrhizobium* and *Pseudomonas* for boosting the production of acidic environment crops.

## Data Availability

The datasets presented in this study can be found in online repositories. The names of the repository/repositories and accession number(s) can be found in the article/Supplementary material.
