# Supplementary material for: Decoding the functional diversity of plant growth-promoting bacterial communities in the soils of Western Ghats, Tamil Nadu, India
Source: Front Microbiol. 2026 Aug 21;17:1865342. doi: 10.3389/fmicb.2026.1865342 (PMC13541767; doi:10.3389/fmicb.2026.1865342)
Supplement: Supplementary file 1 [file Table_1.DOCX]

**Supplementary Table 1** **Assessment of plant growth-promoting rhizobacteria from the soils of Western Ghats**

| **Bacterial isolates** | **Ammonia** | **Phosphorus** | **Potassium** | **Zinc** | **Siderophore** | **HCN** | **IAA** | **Overall score** |
| --- | --- | --- | --- | --- | --- | --- | --- | --- |
| S1B1 | 0 | 0 | 0 | 0 | 0 | 0 | 0 | 0 |
| S1B2 | 0 | 0 | 0 | 0 | 0 | 0 | 0 | 0 |
| S1B3 | 2 | 0 | 0 | 0 | 0 | 0 | 0 | 2 |
| S1B4 | 0 | 0 | 0 | 0 | 0 | 0 | 0 | 0 |
| S1B5 | 1 | 0 | 0 | 0 | 0 | 0 | 0 | 1 |
| S1B6 | 1 | 0 | 0 | 0 | 0 | 0 | 2 | 3 |
| S2B1 | 1 | 0 | 0 | 0 | 1 | 0 | 0 | 2 |
| S2B2 | 1 | 0 | 0 | 0 | 0 | 0 | 0 | 1 |
| S2B3 | 1 | 1 | 3 | 3 | 2 | 0 | 2 | 12 |
| S2B4 | 1 | 0 | 0 | 0 | 0 | 0 | 0 | 1 |
| S2B5 | 1 | 0 | 0 | 0 | 0 | 0 | 0 | 1 |
| S2B6 | 1 | 0 | 1 | 0 | 0 | 0 | 0 | 2 |
| S2B7 | 2 | 1 | 1 | 2 | 0 | 0 | 2 | 8 |
| S2B8 | 1 | 1 | 1 | 2 | 0 | 1 | 1 | 7 |
| S2B9 | 0 | 0 | 0 | 0 | 0 | 1 | 1 | 2 |
| S2B10 | 0 | 0 | 0 | 0 | 0 | 0 | 1 | 1 |
| S2B11 | 0 | 0 | 0 | 0 | 0 | 0 | 1 | 1 |
| S2B12 | 0 | 0 | 0 | 0 | 0 | 0 | 1 | 1 |
| S2B13 | 1 | 0 | 1 | 0 | 0 | 0 | 1 | 3 |
| S2B14 | 1 | 0 | 0 | 0 | 0 | 0 | 1 | 2 |
| S2B15 | 1 | 0 | 1 | 0 | 0 | 0 | 1 | 3 |
| S2B16 | 1 | 0 | 0 | 0 | 1 | 1 | 0 | 3 |
| S2B17 | 1 | 0 | 0 | 0 | 0 | 0 | 0 | 1 |
| S2B18 | 1 | 0 | 1 | 0 | 0 | 0 | 0 | 2 |
| S2B19 | 0 | 0 | 0 | 0 | 0 | 0 | 0 | 0 |
| S2B20 | 1 | 0 | 0 | 0 | 0 | 0 | 0 | 1 |
| S2B21 | 1 | 0 | 0 | 0 | 1 | 0 | 0 | 2 |
| S2B22 | 1 | 0 | 0 | 0 | 0 | 0 | 0 | 1 |
| S3B1 | 2 | 0 | 0 | 0 | 0 | 0 | 0 | 2 |
| S3B2 | 1 | 1 | 1 | 0 | 0 | 0 | 0 | 3 |
| S3B3 | 1 | 0 | 0 | 0 | 0 | 0 | 0 | 1 |
| S3B4 | 0 | 0 | 0 | 0 | 1 | 1 | 0 | 2 |
| S4B1 | 2 | 0 | 1 | 0 | 2 | 0 | 0 | 5 |
| S4B2 | 0 | 0 | 0 | 0 | 1 | 1 | 0 | 2 |
| S4B3 | 0 | 0 | 1 | 0 | 0 | 0 | 0 | 1 |
| S4B4 | 0 | 0 | 0 | 0 | 0 | 0 | 0 | 0 |
| S4B5 | 1 | 0 | 0 | 1 | 0 | 0 | 0 | 2 |
| S4B6 | 0 | 0 | 1 | 0 | 0 | 0 | 0 | 1 |
| S5B1 | 2 | 0 | 1 | 0 | 2 | 0 | 0 | 3 |
| S5B2 | 0 | 0 | 1 | 0 | 0 | 0 | 0 | 1 |
| S5B3 | 1 | 0 | 1 | 0 | 0 | 0 | 0 | 2 |
| S5B4 | 0 | 0 | 0 | 0 | 0 | 0 | 0 | 0 |
| S5B5 | 1 | 1 | 1 | 2 | 2 | 0 | 1 | 8 |
| S5B6 | 1 | 0 | 1 | 0 | 1 | 0 | 0 | 3 |
| S5B7 | 0 | 0 | 0 | 0 | 0 | 1 | 2 | 3 |
| S5B8 | 0 | 0 | 0 | 0 | 0 | 0 | 0 | 0 |
| S6B1 | 2 | 0 | 2 | 0 | 2 | 2 | 3 | 11 |
| S6B2 | 2 | 0 | 0 | 0 | 0 | 0 | 1 | 3 |
| S6B3 | 2 | 0 | 0 | 0 | 2 | 0 | 2 | 6 |
| S6B4 | 3 | 0 | 0 | 0 | 0 | 0 | 1 | 4 |
| S6B5 | 3 | 0 | 0 | 0 | 0 | 0 | 0 | 3 |
| S6B6 | 1 | 0 | 1 | 0 | 0 | 0 | 1 | 3 |
| S7B1 | 1 | 0 | 0 | 0 | 0 | 0 | 0 | 1 |
| S7B2 | 2 | 0 | 0 | 0 | 0 | 0 | 0 | 2 |
| S7B3 | 3 | 0 | 0 | 0 | 0 | 0 | 0 | 3 |
| S7B4 | 3 | 0 | 0 | 0 | 0 | 0 | 1 | 3 |
| S8B1 | 1 | 0 | 0 | 0 | 0 | 0 | 1 | 2 |
| S8B2 | 2 | 0 | 0 | 0 | 0 | 0 | 1 | 3 |
| S8B3 | 1 | 0 | 0 | 0 | 0 | 0 | 0 | 1 |
| S8B4 | 2 | 0 | 0 | 0 | 0 | 0 | 1 | 3 |
| S8B5 | 1 | 0 | 0 | 0 | 0 | 0 | 0 | 1 |
| S8B6 | 1 | 0 | 0 | 0 | 0 | 0 | 1 | 2 |
| S8B7 | 2 | 0 | 0 | 0 | 0 | 0 | 1 | 3 |
| S8B8 | 2 | 0 | 0 | 0 | 0 | 0 | 1 | 3 |
| S8B9 | 2 | 0 | 0 | 0 | 0 | 0 | 1 | 3 |
| S8B10 | 2 | 0 | 0 | 0 | 0 | 0 | 0 | 2 |
| S8B11 | 2 | 0 | 0 | 0 | 0 | 0 | 0 | 2 |
| S8B12 | 3 | 0 | 0 | 0 | 0 | 0 | 1 | 4 |
| S8B13 | 1 | 0 | 0 | 0 | 0 | 0 | 0 | 1 |
| S9B1 | 3 | 0 | 0 | 0 | 0 | 0 | 0 | 3 |
| S9B2 | 2 | 0 | 0 | 0 | 0 | 0 | 1 | 3 |
| S9B3 | 2 | 0 | 0 | 0 | 3 | 0 | 0 | 5 |
| S9B4 | 1 | 0 | 0 | 0 | 0 | 0 | 1 | 2 |
| S9B5 | 2 | 0 | 0 | 0 | 0 | 0 | 0 | 2 |
| S9B6 | 1 | 0 | 0 | 0 | 0 | 0 | 1 | 2 |
| S9B7 | 2 | 0 | 0 | 0 | 0 | 0 | 0 | 2 |
| S9B8 | 2 | 0 | 0 | 0 | 0 | 0 | 0 | 2 |
| S9B9 | 2 | 0 | 0 | 0 | 0 | 0 | 1 | 3 |
| S9H10 | 3 | 0 | 1 | 1 | 2 | 0 | 2 | 9 |
| S9H11 | 2 | 0 | 0 | 0 | 0 | 0 | 0 | 2 |
| S9H12 | 1 | 0 | 0 | 0 | 0 | 0 | 1 | 2 |
| S9H13 | 3 | 0 | 0 | 0 | 0 | 0 | 0 | 3 |
| S9H14 | 1 | 0 | 0 | 0 | 0 | 0 | 1 | 2 |
| S9H15 | 1 | 0 | 0 | 0 | 0 | 0 | 1 | 2 |
| S9H16 | 2 | 0 | 0 | 0 | 0 | 0 | 1 | 3 |
| S10B1 | 1 | 0 | 0 | 0 | 2 | 0 | 0 | 3 |
| S10B2 | 2 | 0 | 1 | 2 | 2 | 0 | 2 | 9 |
| S10B3 | 1 | 0 | 0 | 2 | 2 | 0 | 0 | 5 |
| S10B4 | 1 | 0 | 0 | 0 | 1 | 0 | 0 | 2 |
| S10B5 | 2 | 0 | 0 | 0 | 0 | 0 | 1 | 3 |
| S10B6 | 1 | 0 | 1 | 1 | 2 | 0 | 1 | 6 |
| S10B7 | 2 | 0 | 1 | 0 | 2 | 0 | 0 | 5 |
| S10B8 | 1 | 0 | 1 | 0 | 1 | 0 | 0 | 3 |
| S10B9 | 2 | 0 | 2 | 2 | 0 | 0 | 0 | 6 |
| S10B10 | 2 | 0 | 0 | 0 | 0 | 0 | 0 | 2 |
| S10B11 | 1 | 0 | 0 | 0 | 0 | 0 | 0 | 1 |
| S10B12 | 2 | 0 | 0 | 0 | 1 | 0 | 0 | 3 |
| S10B13 | 2 | 0 | 0 | 0 | 0 | 0 | 0 | 2 |
| S10B14 | 2 | 0 | 0 | 0 | 0 | 0 | 0 | 2 |
| S10B15 | 1 | 0 | 1 | 0 | 0 | 0 | 0 | 3 |
| S10B16 | 2 | 0 | 0 | 0 | 0 | 0 | 1 | 3 |

**Supplementary Table 2 Morphological characterization of** **selected bacterial isolates**

| **Morphological characters** | **S6B1** | **S2B3** | **S9H10** | **S10B2** |
| --- | --- | --- | --- | --- |
| Gram reaction | Negative | Negative | Negative | Negative |
| Pigmentation | Pale white | Pale white | Pale white | Pale white |
| Appearance | Rod-shaped | Rod-shaped | Rod-shaped | Rod-shaped |
| Shape | Entire round | Entire round | Round, serrated | Entire round |
| Size | Medium | medium | Minute, tiny | Small |
| Nature | Translucent | opaque | translucent | Opaque |
| Texture | glossy | smooth | Glossy | Smooth, shiny |
| Elevation | convex | convex | convex | Convex |

**Supplementary Table 3 Biochemical characterization of selected bacterial isolates**

| **Bacterial isolates** | **S6B1** | **S2B3** | **S9H10** | **S10B2** |
| --- | --- | --- | --- | --- |
| Catalase | **+** | **+** | **+** | **+** |
| oxidase | **+** | **+** | **+** | **+** |
| Nitrate reduction | **+** | **+** | **+** | **+** |
| Starch hydrolysis | **+** | **-** | **-** | **-** |
| MR | **-** | **-** | **-** | **-** |
| VP | **-** | **-** | **-** | **-** |
| Indole | **-** | **-** | **-** | **-** |
| Citrate | **+** | **+** | **+** | **+** |
| Urease | **-** | **-** | **-** | **-** |
| H_2_S | **-** | **-** | **-** | **-** |
| Amylase | **+** | **+** | **+** | **+** |
| Glucose | **+** | **+** | **+** | **+** |
| Sucrose | **+** | **+** | **+** | **+** |
| Lactose | **-** | **-** | **-** | **-** |
| Galactose | **-** | **-** | **-** | **-** |
